# Secondary traumatization in refugee care—EMDR intervention for interpreters (STEIN): a study protocol for a quasi-randomized controlled trial

**DOI:** 10.1186/s13063-024-08480-4

**Published:** 2024-10-01

**Authors:** Irja Rzepka, David Kindermann, Hans-Christoph Friederich, Christoph Nikendei

**Affiliations:** Department for General Internal and Psychosomatic Medicine, University Hospital Heidelberg, Heidelberg, Germany

**Keywords:** Secondary traumatic stress, Interpreters, Refugee care, EMDR

## Abstract

**Background:**

By the end of 2022, more than 100 million people worldwide fled their homes. Before, during and after their flight, refugees have high risk of experiencing traumatic events. Accordingly, around every third refugee is affected by posttraumatic stress disorder. For adequate mental health care, the service of interpreters is often urgently needed to overcome existing language barriers. However, repeated exposure with details of traumatic narratives, as experienced by interpreters, can be burdensome and can lead to trauma sequela symptoms in terms of secondary traumatic stress. Only few studies have examined the treatment of secondary traumatic stress to date. Based on the recommendations for the treatment of posttraumatic stress disorder with confrontational methods, this study was designed to evaluate the effectiveness of an eye movement desensitization and reprocessing (EMDR) intervention in a sample of interpreters working in refugee care suffering from secondary traumatic stress symptoms.

**Methods:**

To evaluate the effectiveness of an EMDR intervention for the treatment of secondary traumatic stress symptoms, a quasi-randomized controlled trial using a waiting group design will be performed. Participants will be treated with a maximum of 6 sessions based on EMDR standard protocol. Primary outcome is the symptom load of secondary traumatic stress, assessed with the Questionnaire for Secondary Traumatization, while secondary outcomes comprise further symptom complexes such as PTSD due to self-experienced traumatic events, depression, anxiety, and somatization as well as quality of life, quality of professional life, and psychological wellbeing that will be assessed with the PDS, PHQ-9, GAD-7, SSD-12, SF-12, PROQOL-5, and WHO-5, respectively.

**Discussion:**

Our primary interest is to determine the efficacy of an EMDR intervention in interpreters affected by secondary traumatic stress, especially how many sessions are needed for significant symptom reduction. Change of associated symptom complexes and quality of life will be investigated. Reprocessing one’s own stressful experiences may also contribute to this, which is not the focus of the treatment but relevant to the EMDR protocol. This study aims to assess if EMDR could be an acceptable, effective, and time-efficient method for reducing work-related secondary traumatization.

**Trial registration:**

German Clinical Trials Register, DRKS00032092, registered 16 June 2023.

## Background

In 2022, more than 100 million people worldwide had fled their home or place of origin due to war, natural disasters, or the consequences of poverty [1]. Of these, more than 6 million people were from Ukraine alone [2]. It is commonly known that the prevalence of mental illnesses of refugees and asylum seekers is far higher than in the general population due to stressors occurring pre-, peri-, and post-flight [3–5]. In particular, posttraumatic stress disorder (PTSD) affects around 30% of the refugee population [3]. However, the number of people having exposed traumatic events is assumed to be substantially higher [6, 7].

Mental health care of refugees largely depends on the availability of interpreters, as, especially in the early post-migration phase, the language barrier is a significant barrier in implementing adequate mental health care. During interpreters’ work, interpreters are often confronted with personal narratives of refugees, including traumatic events. Empathetic listening gives rise to a simultaneous experience of vivid imagination and somatosensory sensations. These narratives can affect the listening person but can also be extremely burdensome. This empathic compassion can even result in symptoms similar to posttraumatic stress disorder that are comprised of intrusions, avoidance behavior, hyperarousal, and alteration of cognition and mood even though the event was not personally experienced. This phenomenon is described as secondary traumatization [8]. The risk to develop symptoms of secondary traumatic stress can increase not only by the quantity of reports of traumatic events but also by the way in which the experience is narrated [9, 10]. Similar concepts that overlap with secondary traumatic stress are compassion fatigue that is characterized by feelings of helplessness, psychological and emotional exhaustions [11], and vicarious traumatization that includes a permanent change in cognitive schemas and beliefs [12]. To take this into account, the diagnostic criteria for post-traumatic stress disorder have been adapted in the DSM-V: in the recent version, “experiencing repeated or extreme exposure to aversive details of the traumatic event(s)” is also sufficient as an A-criterion [13]. In ICD-10 and ICD-11, on the other hand, a traumatic event is defined as an event of “extremely threatening or horrific nature,” that is directly experienced or witnessed [14], and thus rather broad, which stems from the observation that PTSD symptoms can develop after experiencing a traumatizing event of any kind and that the definition of a traumatic event is therefore not decisive [15]. Research has shown that PTSD symptoms can develop after secondary traumatization, but to a lesser extent [16]. However, an independent diagnosis for symptomatology due to secondary traumatization cannot be found in either of the two diagnostic manuals.

Regarding the prevalence of secondary traumatic stress, Kindermann et al. [17] showed that in a sample of interpreters working in refugee care, 21% are affected by trauma sequela symptoms due to secondary traumatization, and around 6% show high symptom scores. However, this is not the only population that can be affected by secondary traumatization. Secondary traumatic stress is particularly prevalent among trauma therapists and other caregivers of traumatized clients [8, 18], nurses [19], or first responders [20] as highlighted in various studies.

However, despite the traumatic stress burden resulting from working with traumatized patients, the knowledge about effective treatment is rather scarce. In a systematic review on the treatment of secondary traumatic stress among mental health workers conducted by Bercier and Maynard in 2015 [21], none of the selected studies met the inclusion criteria. The authors concluded that insufficient attention had been given to the distinction of existing concepts and the evaluation of the necessity for large-scale intervention in the field. This gap was attributed to the ongoing development of research, which primarily focused on genesis and clinical presentation. In 2012, Sprang et al. [22] presented an overview of the current state of knowledge as well as the challenges with regard to the research field of secondary traumatic stress. With regard to therapeutic interventions, they showed that various symptom-related approaches have been published, such as psychoeducation, emotion regulation training, or mindfulness training. With a view to improving interventions for secondary traumatic stress, two different approaches for further research were recommended: the amelioration of preventive interventions and specific interventions for affected workers.

Due to symptoms having a clinical similarity to posttraumatic stress disorder, it is suspected that interventions based on therapeutic approach for PTSD can be equally effective for secondary traumatic stress [22]. In national and international guidelines, exposition therapy approaches as first line treatment for posttraumatic stress disorder are recommended [23–27]. Among others, eye movement desensitization and reprocessing (EMDR) is a validated and effective treatment for PTSD [28]. While the underlying effect of bilateral eye stimulation is not fully understood [29], there is large evidence of EMDR effectively reducing symptoms of traumatic stress [30]. The planned study will examine the effectiveness of EMDR on secondary traumatic stress symptoms in a sample of interpreters working in refugee care. Therefore, a quasi-randomized controlled trial will be conducted, with a waitlist-control group to compare the development of symptoms of secondary traumatic stress in each group with and without EMDR intervention. The aim is to examine the hypothesis that EMDR intervention significantly reduces symptom burden of secondary traumatization compared to waitlist control.

## Methods/design

To answer the research question if EMDR is more effective in reducing symptoms of secondary traumatization in adult interpreters caused by their work in refugee care settings than in a waitlist-control group, a quasi-randomized controlled trial (RCT) with an EMDR intervention of three to six sessions will be conducted. Primary outcome is the symptom burden of secondary traumatic stress symptoms measured with questionnaire for Questionnaire for Secondary Traumatization (“Fragebogen zur Sekundären Traumatisierung,” FST) [31]. Secondary outcomes are symptom burden of PTSD due to self-experienced traumatic events (assessed with Posttraumatic Diagnostic Scale [32]), depression (PHQ-9 [33]), anxiety (GAD-7 [34]), and somatization (SSD-12 [35]) as well as quality of life (SF-12), quality of professional life (PROQOL-5 [36]), and psychological wellbeing (WHO-5 [37]). The study is conducted with the aim of improving trauma-informed care and, more specifically, expanding the knowledge of trauma-confrontation techniques in the broader context of trauma-related disorders, as these have repeatedly proven to be effective [38, 39].

### Participants and recruitment

**Fig. 1:**
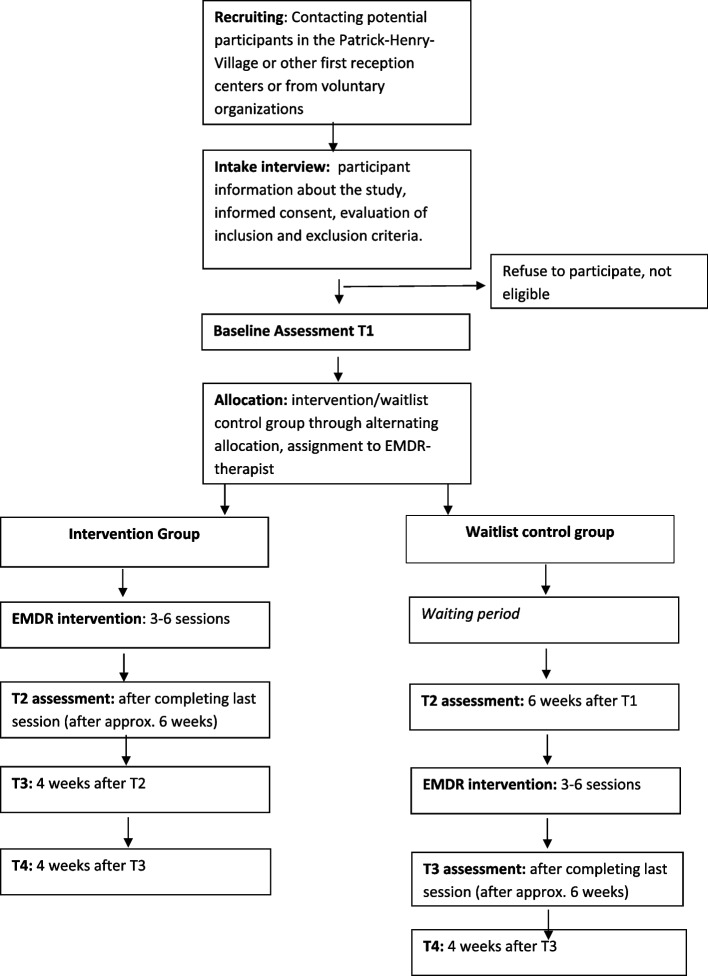
Timeline of the study design

Potential participants will be recruited in a German State registration and reception center for refugees in Heidelberg (Patrick-Henry-Village). In this initial reception center, interpreters work as professionals or on a voluntary basis in various settings, such as in social or procedural advice or in the medical field, in order to include different types of workload, intensity, and forms of care for refugees [17]. In addition, further interpreters are to be recruited in other initial reception settings and from charitable organizations. They will be contacted and informed by our study team. If they are interested, they will be invited to an initial screening appointment and informed verbally and written about the procedure of the study. If they agree to participate in the study, written informed consent will be obtained. A personal evaluation to assess inclusion and exclusion criteria will be conducted by an experienced therapist of the study team. Inclusion criteria are adult participants (≥ 18 years), working on professional or voluntary basis as interpreters in the Patrick-Henry-Village or other settings that suffer from secondary traumatic stress, verified using the Questionnaire for Secondary Traumatization and reaching the cut-off-score of ≥ 65 [31]. Exclusion criteria for participation in the study are active suicidal ideation or self-harming behavior, acute substance abuse, and psychotic symptoms. For this purpose, the respective symptoms and substance use behavior are asked about in detail during the personal interview, but no standardized tool is used. The timeline of the study design is depicted in Fig. 1. Additionally, a questionnaire-based survey with a set of different questionnaires for secondary outcomes will be conducted. These data will be assessed with an online tool. The detailed SPIRIT checklist can be viewed as an enclosed document.

### Primary outcome measure

The questionnaire-based data collection will be carried out upon the initial screening appointment (T1) for each group. For the intervention group, the second data collection point (T2) is around 6 weeks after T1, after the last session for the intervention, then 4 weeks (T3) and 8 weeks (T4) after T2. For the waitlist control group, T2 will be 6 weeks after T1, then after their last session (T3) and 4 weeks (T4) after T3. Also see Fig. 1. The participants will receive the link to the online questionnaires as well as reminders, if necessary, by email.

Secondary traumatic stress will be surveyed with the Questionnaire for Secondary Traumatization (“Fragebogen zur Sekundären Traumatisierung,” FST) [31]. This questionnaire surveys different symptoms of secondary traumatic stress based on diagnostic criteria of DSM-V for posttraumatic stress disorder, such as rumination, intrusions or nightmares, avoidance behavior, and, furthermore, cognitive or behavioral changes. The frequency of the above symptoms in the last week is assessed by 31 items on a 5-point Likert scale (1 = never, 5 = very often). From a sum score of ≥ 65, moderate secondary traumatic stress can be assumed. The structure of the questionnaire is similar to the Impact of Event Scale [40] and is designed to be used in professional context. It showed good internal consistency for the total score (Cronbach’s *α* = 0.94) in three different samples of professionals.

### Secondary outcome measure

#### Posttraumatic stress

According to current knowledge, the development of secondary traumatic stress is positively correlated with one’s own biography and the experience of traumatic event, with varying correlations [9]. For the procedure of EMDR treatment, it is important to know about traumatic events in the past and potential accompanying symptoms. Therefore, a questionnaire to assess primary traumatization (Posttraumatic Diagnostic Scale, PDS [41]) was added. The current version based on DSM-5 criteria will be used [32, 42]. The PDS-5 is a self-reporting questionnaire which measures symptoms of PTSD with 20 items on a 5-point Likert scale (0 = not at all, 4 = 6 or more times a week/severe) and is a valid and reliable measure with sensitivity of 89%, specificity of 75%, and a high internal consistency (Cronbach α = 0.92). A sum score of ≥ 36 indicates a probable diagnosis of PTSD [42].

#### Professional quality of life

Due to the related concepts to secondary traumatic stress caused by professional activity, a questionnaire for “Professional Quality of Life” will be assessed. The PROQOL-5 inquires with 30 questions factors of compassion satisfaction, compassion fatigue, symptoms of burnout, and secondary traumatic stress. On a 5-point Likert scale (0 = never, 5 = very often), participants will be asked about different experiences related to their professional work as a translator. The scale has been used in different professional contexts and shows high internal consistency for all subscales (Cronbach’s *α* = 0.90 for compassion satisfaction scale, *α* = 0.80 for the burnout scale, and *α* = 0.82 for secondary traumatic stress scale). With a mean sum score for any scale of 50 (SD: 10), a sum score ≥ 57 on any scale can give an indication of elevated strain in this area [36, 43].

#### Depression and anxiety

In a study by Živanović et al. [10] secondary traumatic stress, especially negative alteration of cognition, mood, and reactivity, was shown to influence depressive and anxiety symptomatology. Therefore, depressive symptoms will be assessed by the Patient Health Questionnaire, PHQ-9 [33]. It consists of 9 items that can be rated on a 4-point Likert scale (0 = not at all, 3 = almost every day). A score of ≥ 10 showed high sensitivity (88%) and specificity (88%) for the presence of a major depressive episode. The questionnaire shows excellent reliability (Cronbach’s α = 0.86–0.89). Anxiety symptoms will be surveyed with General Anxiety Disorder, GAD-7 [34]. It is comprised of 7 items asking for typical symptoms of generalized anxiety disorder. Equally, symptoms can be equally quantified on a 4-point Likert scale. A cut-off-value of ≥ 10 shows with high sensitivity and specificity of 0.8 likeliness of generalized anxiety disorder. Reliability is likewise excellent (Cronbach’s α = 0.92).

#### Somatic distress

To date, there is sparse data on somatic symptoms in secondary traumatic stress [44]. However, it is known that there is a high prevalence of somatic symptoms in PTSD [45] and somatic complaints are also a common symptom of burnout [46]. Therefore, a questionnaire on somatic symptoms is also collected for the present study. The scale for Somatic Symptom Disorder (SSD-12) does assess B criteria of somatic stress disorder, i.e., the perception of symptom-related thoughts, feelings, and behaviors on a scale from 0 = never to 4 = very often. A sum score value of ≥ 23 shows an increased risk of somatic stress disorder. The questionnaire shows high reliability (Cronbach’s α = 0.94) [35, 47].

#### Quality of life

Beyond the previous aspects, health-related quality of life (Short Form 12, SF-12 [48]) as well as psychological wellbeing (WHO-5 [37]) will be examined. The SF-12 questionnaire is a non-disease-specific instrument and covers 8 categories, which include questions about limitations in physical or social activities, hindrance of usual role activities due to physical or psychological problems, physical pain, general mental health, vitality, and general health perception. Two scales are used to measure physical (Physical Component Summary, PCS)) and psychological aspects (Mental Component Summary, MCS)) of health-related quality of life, which show a good internal consistency with Cronbach’s *α* = 0.89 (PCS) and *α* = 0.89 (MCS) [49, 50]. Summary scores for each scale are calculated, with an average ≥ 50 indicating a better mental or physical health than the average population and ≤ 50 indicating a worse mental or physical health than the average population. The WHO-5 is one of the most frequently used questionnaires to measure general, subjective well-being with a good internal consistency (Cronbach’s *α* = 0.80–0.92). Psychological well-being is recorded using five questions about a good mood, inner peace and relaxation, energy, ability to regenerate through sleep, and enthusiasm in the last 2 weeks. These can be agreed on a scale of 1–5 with 0 = at no time and 5 = all the time. A total score is calculated based on the number of points and multiplied by 4, with a score of 0 representing the worst and a score of 100 representing the best well-being. Used as a screening tool for depression, a sum score ≤ 50 indicates a positive screening result [37]. For the analysis of the results, the changes in the total scores or the described subscales of the primary and secondary outcomes before the intervention (T1) compared to after the intervention (T2, T3), as well as in the comparison of the intervention group with the control group, are of interest.

### Randomization

**Fig. 2:**
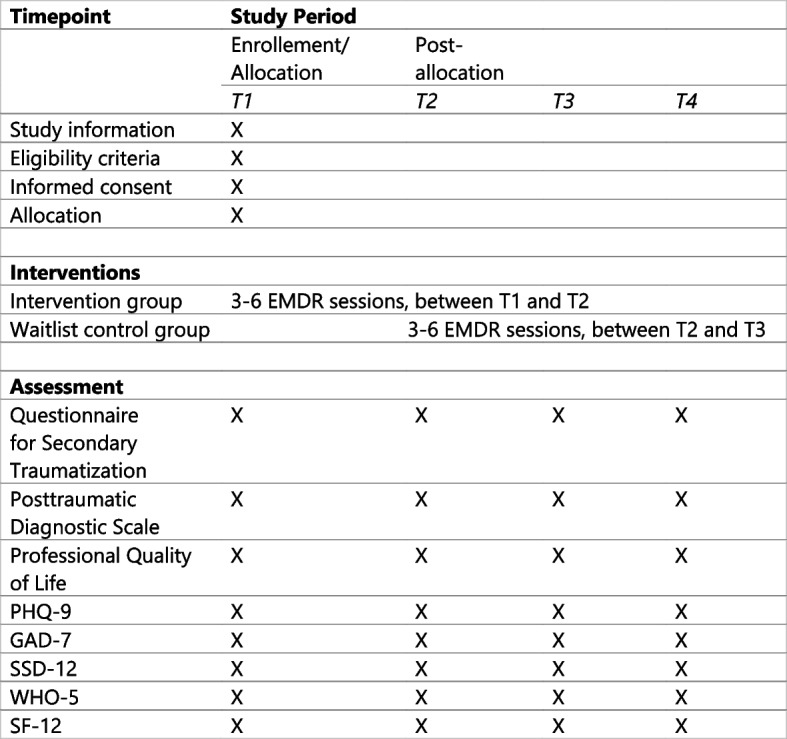
Participant timeline

Participants will be randomly assigned in either the intervention group or waiting list control group. Because of a rather small sample size, and to ensure seamless and reliable allocation for available therapists, this will be carried out through alternating allocation of participants at the initial screening interview for the study to avoid large differences in sample size. The allocation to one of the two groups is therefore already determined before inclusion in the study. Participants will be informed verbally at the time of the initial screening interview by the responsible therapist. Blinding is neither possible for the participant (as filling out of questionnaires during waiting time), nor for the EMDR therapist, as collaborative planning is necessary for the distribution of participants, as the therapists mainly work clinically. The team of EMDR therapists available to conduct the intervention comprises of five persons. Both groups will receive the same intervention that is planned as follows. A detailed participant timeline can be found in Fig. 2.

### Intervention

Participants will be allocated to a therapist, medical doctor or psychologist of the department who will have completed, at least, EMDR training level 1. The EMDR treatment that will be carried out is comprised of 8 phases according to Shapiro [51]. In the first session, phases 1 and 2 will be conducted. The first phase includes the establishment of a therapeutic relationship as well as the exploration of the symptomatology and the trauma history. In this case, the therapist will comprehensively examine the symptoms of secondary traumatization including distressing situations relating to their work in refugee care in the present. Emotion regulation strategies of the participant will be elaborated. With help of the “adaptive information processing” model (AIP), the therapist and the participant will work out underlying stress situations from the professional context. According to the AIP model, traumatic memories, but also other “live events” or disturbing experiences that do not accord to the trauma criteria, can create a “pathogenetic memory.” These pathogenetic memories are stored in a dysfunctional way, are not connected to adaptive memory networks, and, subsequently, cause symptoms. Based on this assumption, repeated exposure to details of refugees’ traumatic experiences leads to dysfunctional storage of this experience of the affected interpreters [52, 53]. The therapist and the participant will carve out the respective distressing situations and classify them on a scale from 0 to 10 (subjective level of disturbance, SUD), as stated in the EMDR manual. Additionally, the participant will be introduced to the EMDR technique itself. An imaginative exercise, “safe place,” will be executed together and reinforced with EMDR technique. Techniques to stop flashbacks will be discussed. Additionally, the participant will receive a further imagination exercise, called “safe exercise,” that helps distancing oneself from disturbing thoughts. The first session takes 50–60 min. Phase 3–7 will be conducted in the following sessions according to the EMDR standard protocol. Phase 8 will be performed at the beginning of every exposition session. The EMDR standard protocol will be applied. This means that the disturbing memories will be worked on in a specific (“the first, the worst, the last”). However, by mutual agreement, the order in which the stressful memories are processed is left open to the therapist and the participant, if this leads to greater openness to therapy and adherence. Sessions will take place once a week; the therapist and participant will agree mutually on the date of the sessions, and they can be adapted to the participants’ needs. The confrontational sessions ends ideally when the SUD is 0. However, depending of the processed content, this cannot always be achieved, for example if the participant processes earlier stressful memories through so-called affect bridges. In this case, the session can be closed if SUD is not 0 but only reduced from the initial level. In this case, work on this memory will continue in the following session (phase 8). The processing time can differ between patients and takes around 60–90 min. A maximum of six sessions will be conducted. The number of sessions will be adapted to the participants needs. This implies that within the span of these six sessions, the stressful situations related to the participant’s work with refugees, which the participant seeks to address, are systematically managed. If a complete reduction of the SUD is not achieved within a single session, the issue may be resolved over 2–3 sessions. However, it is generally expected that each session is self-contained, as the nature of the underlying stressors suggests that a full reduction in SUD can be accomplished within one session. Consequently, the number of sessions corresponds to the number of specific situations requiring attention. Conducting fewer than six sessions does not imply that any therapeutic content is omitted. Participants in both groups receive the same intervention.

### Monitoring

The responsible research team members meet once a week to monitor the process of the study, the data collection, and identification of need for adjustment. Additionally, the conducted EMDR interventions will be supervised during the respective execution. No interim analysis will be performed. There are no stopping guidelines for premature termination of the study.

### Concomitant care, serious adverse events

Due to ethical considerations, participants can receive psychotherapeutic treatment or psychopharmacological medication during the study. The existence of simultaneous psychotherapy or psychopharmacological medication will be surveyed in every data collection point so that any changes in this regard are recorded. By conducting the study, no serious adverse events (SAE) are expected. Cases will be supervised regularly by an external EMDR supervisor. However, if in any case of a serious adverse event happens, this will be immediately and directly reported contact to the study team and the principal investigator. Serious adverse events include suicidal ideation, severe self-harm, or severe and ongoing exacerbation of the symptoms of secondary traumatization in the context of EMDR treatment. These will be reported systematically in non-standardized language as well as discontinuation of the study participations for other reasons. Responsible therapists will be informed about this procedure prior to the study. It will be reported by Serious Adverse Event Report Form [54]. Handling of adverse events will be decided individually. Therapeutic treatment through the outpatient clinic of the department is possible if required. A short-term increase in psychological stress is inherent in the EMDR procedure, as it is a trauma confrontation procedure. This normally subsides within a few days. This is not systematically reported as harm, as it could arise as part of the treatment, but will be discussed in the joint sessions with the responsible therapist.

### Statistical analyses

The quantitative data will be monitored and analyzed by the study team and colleagues of the research group using SPSS [55]. As the statistical evaluations are carried out by the study team with the support of the biostatistician, blinding is not performed. Baseline characteristics will be compared to assessed differences of the intervention and control group, especially regarding the symptom load of secondary traumatization symptoms as well as on the presence of self-experienced traumatic events and PTSD symptoms. The effectiveness of the EMDR intervention will be assessed using a piece wise growth curve model [56], a type of the latent growth curve model, based on the assumption that change of symptomatology along the study duration is not linear but depends to certain change points. In this model, growth rates between different time periods are compared. The main criterion of interest is the symptom load of secondary traumatic stress assessed with the FST. Further parameters to be analyzed are symptom load of depression, anxiety, and somatic distress as well as professional quality of life and quality of life. For the data collected, time periods of interest are before and after the intervention for intervention group (T1–T2) and waitlist control group (T2–T3), after the intervention to follow up for both groups (intervention group T2–T3/T4, control group T3–T4), and before and after the waiting period for the control group (T1–T2). Covariates will include current psychiatric treatment, current psychopharmacological prescription or medication change, and personal traumatic experience. The data of all randomized participants who filled out the pre-and post-questionnaires and, from the intervention group, who completed at least two sessions (one preparatory session with investigation of the experiences that will be processed (phases 1–2) and at least one confrontational session (phase 3–7/8) will be included in the analysis (per-protocol analysis). Reasons for incomplete participation in the intervention will be presented descriptively. With regard to the calculation of the sample size for a piece wise growth model, there is only scarce literature on how to do so [57]. Therefore, the sample size is calculated on the basis of analysis of variance (ANOVA) with repeated measures. According to literature, EMDR shows large effect sizes (*g* = 1.01 (CI 95% 0.42 to 1.62). For the present study, however, the effect size of EMDR on secondary traumatization is not known yet. Therefore, a conservative calculation with a medium effect (*d* = 0.5) was carried out. Assuming a type I error of *α *= 0.05 and a statistical power of 0.8, a sample size of *n *= 34 is calculated [58]. Assuming a drop-out rate of 18% [59], a sample size of 40 is aimed for. Missing data will be first identified and analyzed. No further measures will be carried out in case of missing data < 5%. If > 5% of the data are missing, it will be examined whether these are related to certain variables, such for example with symptom burden and whether patterns can be recognized. If these data are missing at random, no further procedures will be carried out. If missing values are systematically related to one of the recorded variables, a multiple imputation can be performed [60]. Due to a lack existing studies, the distribution of the data cannot be estimated. Therefore, the collected data is first checked for normal distribution and if not normally distributed transformed in order to carry out the analyses with transformed data.

### Data collection management

Data will be collected manually at the first appointment (informed consent, Questionnaire for Secondary Traumatization, information on exclusion criteria) and then via online-survey with “Unipark” [61]. Correct and accurate data collection and, if necessary, reminding of participants will be performed manually at regular intervals. No further control of data collection will be performed. Only members of the study team and colleagues of the research group have access to the dataset. Data are stored pseudonymized in a secure and accessible manner in the responsible department. Third parties do not have access to the data.

### Ethics and dissemination

The study is approved by the Ethical Committee of Heidelberg University (S-028/2023). Any substantial changes that influence the conduct of the study will be the discussed in the research team, and a formal amendment of the ethics committee will be examined prior to implementation. All participants will be informed verbally and written about the procedure of the study, including randomization, as well as benefits and possible disadvantages at the time of inclusion by a research team member. They will be informed that they can withdraw from the study at any time. Participants will only be included if they give their written consent. The results of this study will be published after evaluation in a peer-reviewed international journal; besides, no further use of the collected data is planned. Findings will also be presented in relevant research conferences or in local academic context.

## Discussion

In this study, the effectiveness of EMDR intervention in interpreters working in refugee care who are suffering from symptoms of secondary traumatization will be evaluated. The effect on further symptomatology as well as quality of life, quality of professional life, and psychological wellbeing will be evaluated.

EMDR is an effective approach to reduce posttraumatic stress symptoms and is one of the recommended treatments for PTSD [23, 62–64]. However, the primary target of “pathological memory” that will be processed is not self-experienced traumatic events but traumatization through exposure with traumatic details of the refugee’s narratives and empathetic indirect experiencing. It is known that there is a positive correlation of having directly experienced traumatic events and of developing symptoms through secondary traumatization [9]. In a sample of interpreters working in refugee care, 58% experienced traumatic experiences themselves [17]. In EMDR treatment, potential linkages between the secondary traumatic stress and the interpreter’s potential own distressing or traumatic experiences can be activated through the reprocessing. Reprocessing potentially underlying stressful experiences is inherent in the EMDR protocol and could also affect the parameters examined. For the present study, several points of interest will be examined. Firstly, it is crucial to assess whether this type of structured and targeted treatment offer meets the needs of those affected and is subsequently utilized. Furthermore, the study aims to determine the number of sessions required to achieve a significant symptom reduction. EMDR has the potential to be an effective and time-limited intervention for the treatment of work-related secondary traumatization. If the assumption is confirmed, further studies could be carried out in other target populations affected by secondary traumatic stress. Additionally, the effect of emotional bridging to address related personal traumatic experiences will be investigated. Instead of being quantifiable, these results will be observed and stated descriptively after being seen during therapy sessions.

A limiting factor could be the insufficient number of participants. This, indeed, has already been reported to be one of the limiting factors in previous studies that attempted to show the effect of interventions on secondary traumatic stress [21]. However, this will be addressed by individually scheduling the sessions and adapting the frequency of sessions to the needs of the participants. In addition, a financial incentive is offered for participants after completing the final questionnaire.

## Trial status

The described trial is according to the protocol version no. 2, 16.10.2023. At the time of the submission of the protocol, the recruitment has started; *n* = 1 participant has been included. Recruitment will be completed approximately in June 2024.

## Data Availability

The final trial dataset will be only accessible for study team.

## Abbreviations

ANOVA: Analysis of variance
AIP: Adaptive information processing
EMDR: Eye movement desensitization and reprocessing
PTSD: Posttraumatic stress disorder
SAE: Serious adverse event
